# Colitis-Mediated Dysbiosis of the Intestinal Flora and Impaired Vitamin A Absorption Reduce Ovarian Function in Mice

**DOI:** 10.3390/nu15112425

**Published:** 2023-05-23

**Authors:** Ze Li, Chengzhen Chen, Wenjie Yu, Lingxia Xu, Haitao Jia, Chen Wang, Na Pei, Zibin Liu, Dan Luo, Jun Wang, Wenfa Lv, Bao Yuan, Jiabao Zhang, Hao Jiang

**Affiliations:** 1 Department of Laboratory Animals, Jilin Provincial Key Laboratory of Animal Model, Jilin University, Changchun 130062, China; lize20@mails.jlu.edu.cn (Z.L.); chencz@jlu.edu.cn (C.C.); ywj22@mails.jlu.edu.cn (W.Y.); lxxu21@mails.jlu.edu.cn (L.X.); jht22@mails.jlu.edu.cn (H.J.); wang_chen22@mails.jlu.edu.cn (C.W.); peina21@mails.jlu.edu.cn (N.P.); liuzb20@mails.jlu.edu.cn (Z.L.); luodan19@mails.jlu.edu.cn (D.L.); yuan_bao@jlu.edu.cn (B.Y.); 2 College of Animal Science and Technology, Jilin Agricultural University, Changchun 130118, China; junwang@jlau.edu.cn (J.W.); lvwenfa@jlau.edu.cn (W.L.)

**Keywords:** colitis, intestinal flora, ovary, steroid hormone, vitamin A

## Abstract

Changes in the composition and ratio of the flora during colitis have been found to potentially affect ovarian function through nutrient absorption. However, the mechanisms have not been fully explored. To investigate whether colitis-induced dysbacteriosis of the intestinal flora affects ovarian function, mice were given dextran sodium sulfate (DSS) through drinking water. High-throughput sequencing technology was used to clarify the composition and proportion of bacterial flora as well as gene expression changes in the colon. Changes in follicle type, number, and hormone secretion in the ovary were detected. The results showed that 2.5% DSS could induce severe colitis symptoms, including increased inflammatory cell infiltration, severe damage to the crypt, and high expression of inflammatory factors. Moreover, vitamin A synthesis metabolism-related genes *Rdh10*, *Aldh1a1*, *Cyp26a1*, *Cyp26b1*, and *Rarβ* were significantly decreased, as well as the levels of the steroid hormone synthase-related proteins STAR and CYP11A1. The levels of estradiol, progesterone, and Anti-Mullerian hormone as well as the quality of oocytes decreased significantly. The significantly changed abundances of *Alistipes*, *Helicobacter*, *Bacteroides*, and some other flora had potentially important roles. DSS-induced colitis and impaired vitamin A absorption reduced ovarian function.

## 1. Introduction

Colitis is a common disease among animals and is related to the feeding process. Animal enteritis can be caused by eating rotten or moldy feed, drinking unclean water, living in poor sanitary conditions, and contracting bacterial infections. Colitis can cause clinical symptoms such as weight loss, diarrhea, and blood in stool and can lead to the decrease of milk production, a lower feed conversion rate, and slow growth [1,2]. The occurrence of colitis is often associated with dysbiosis of the intestinal flora and disruption of the balance between commensal and potentially pathogenic microorganisms in the host, resulting in reduced intestinal flora diversity [3]. Currently, dextran sodium sulfate (DSS) is widely used in constructing animal ulcerative colitis models. DSS damages intestinal epithelial cells, causing dysbiosis; the consequent activation of immune cells leads to intestinal inflammation. Animals that drank water with DSS showed weight loss, hemorrhagic diarrhea, and typical bacterial infectious inflammation [4,5,6].

The intestinal flora is essential for maintaining mammalian health [7,8]. Changes in the intestinal flora lead to changes in the metabolites of microbes, which are important for the health of the host. The intestinal flora can produce short-chain fatty acids (SCFAs) through complex metabolism; SCFAs have anti-inflammatory effects and maintain the health of the intestine [9]. In female animals, chronic inflammation induced by dysregulated intestinal flora ecology induces hyperactivation of primordial follicles in the ovary [10]. With the development of gonad–gut axis-related research, metabolic communication across the gonads and intestinal flora is considered to be physiologically important [11]. In male animals, DSS induced colitis and caused dysbiosis of the intestinal flora and an increase in harmful bacteria. Lipopolysaccharides (LPS) produced by harmful bacteria enter the testis due to circulation and cause inflammation of the testis and epididymis [12,13]. At the same time, intestinal flora dysbiosis also causes disruption of vitamin A absorption, which affects spermatogenesis and reduces sperm quality [14,15]. A study also showed that intestinal flora disorders are associated with the disruption of vitamin A absorption, which affects spermatogenesis and reduces sperm quality [14,15]. Increasingly, it has been shown that animals with ulcerative colitis have low nutritional utilization and suffer from malnutrition, especially impaired absorption of vitamin A [16,17] and disruption of bile acid homeostasis [18,19], which ultimately leads to reduced fertility in animals [20].

At present, there is no sufficient evidence to confirm the effect of DSS-induced co-litis on small intestinal absorption function. It is undeniable that the digestion and ab-sorption of vitamin A mainly occurs in the small intestine. However, the colon can also absorb vitamin A [21]. The main form of vitamin A in the body is retinoic acid, and the formation of retinoic acid requires the catalysis of multiple enzymes. First, intracellular retinol (a vitamin A derivative) binds to cellular retinol binding protein 1 (CRBP1) and forms retinaldehyde in the presence of retinol dehydrogenase (RDH10), and retinal forms retinoic acid under the action of aldehyde dehydrogenase (ALDH1A1). Retinoic acid binds to CRABP1 and acts on the retinoic acid receptor (RARβ), which is then degraded by CYP26A1 [22,23]. Vitamin A promotes the differentiation of spermatogonia [24] and has regulatory effects on follicle development, ovarian steroidogenesis, oocyte maturation, and luteal formation [25]. The main site of vitamin A absorption is in the small intestine, but colonic microorganisms can use crude fiber to produce β-carotene (a vitamin A supplement) that is absorbed by the colon [21]. Several studies have confirmed that vitamin A deficiency hinders follicle development and reduces oocyte quality and ovarian steroid hormone secretion [24]. The ovary is one of the reproductive organs of mammals and an important site for follicle development and steroid hormone production. After sexual maturation, cyclic follicle recruitment and development are regulated by gonadotropins and steroid hormones secreted by the ovaries themselves [26]. Two steroid hormones, namely estrogen and progesterone, are influenced by vitamins [27,28,29]. However, it is not clear whether the disturbance of the intestinal flora in the colon and the development of ulcerative colitis can cause disturbances in vitamin A absorption and metabolism and thus affect ovarian function.

To investigate the effect of colitis on ovarian function, DSS was used to construct a mouse colitis model and clarify the changes in the mouse intestinal flora under DSS induction by 16S rRNA high-throughput sequencing. Subsequently, the potential relationship between vitamin A metabolism-related genes and specific flora was explored by combined transcriptome sequencing analysis. Finally, ovarian reserve, oocyte quality, estradiol (E_2_), progesterone, and anti-Mullerian hormone (AMH) levels were examined. This provides a new perspective for exploring the effects and mechanisms of intestinal flora dysbiosis caused by ulcerative colitis on the reproductive capacity of female animals, especially ovarian function.

## 2. Materials and Methods

### 2.1. Animals

Seven-week-old female BALB/c mice (Liaoning Changsheng Biotechnology Co., Ltd., Benxi, Liaoning, China) were housed at the Experimental Animal Center of Jilin University (Jilin University, Changchun, China). The ambient temperature was controlled at 22 ± 2 °C, the humidity was maintained at 60 ± 5%, and the light/dark cycle was 12 h. One week of pre-feeding was performed before the formal experiments. Mice had unlimited access to standard food and drinking water.

### 2.2. Colitis Model Construction

A mouse colitis model was constructed according to a previously described method [30]. Mice were randomly divided into the NC group and DSS group (30 mice in each group; 5 mice in each cage). Mice in the NC group were given normal drinking water, and mice in the DSS group were given 2.5% DSS (MP Biochemicals, Solon, OH, USA) in drinking water for 7 days. The body weight, fecal condition, and anal bleeding of the mice were recorded daily. Mice were euthanized on day 8 after continuous feeding with water with or without DSS. The colon and contents were then collected, placed in liquid nitrogen, and snap frozen; finally, all samples were stored at −80 °C until further analysis.

### 2.3. DAI Score Assessment

Throughout the experimental period, the body weight of the mice was recorded every morning, and the feces were collected to observe viscosity and bleeding. Fecal bleeding was detected using the Fecal Occult Blood Kit (Zhuhai Beso Biotechnology Co., Ltd., Zhuhai, Guangdong, China). The disease activity index (DAI) score was assessed based on previously described methods [31]. The weight loss, fecal condition, and fecal bleeding were calculated. Details are shown in Supplementary Table S1.

### 2.4. Histological Analysis

The collected colonic tissues were rinsed with PBS, aspirated of excess fluid, and then immediately fixed in 4% paraformaldehyde for 24 h, followed by dehydration and paraffin embedding. Sections (4 μm thick) were stained with hematoxylin and eosin (H&E). Histological scoring was performed according to previous criteria [32].

### 2.5. Vitamin A, E_2_, Progesterone (P), and AMH Level Measurement

Colonic and ovarian samples were obtained according to a previously described method [33]. In brief, colon and ovarian tissues were homogenized in precooled PBS. The homogenates were centrifuged at 5000× *g* for 5 min, and the supernatants were removed. Vitamin A levels in colon and ovarian tissues were measured using a commercially available mouse vitamin A ELISA kit (Shanghai Enzyme-linked Biotechnology Co., Ltd., Shanghai, China), and the vitamin A levels in colon and ovarian tissues were normalized to the tissue weight. The levels of E_2_, P, and AMH in serum were measured by related commercial mouse ELISA kits (Shanghai Enzyme-linked Biotechnology Co., Ltd., Shanghai, China).

### 2.6. 16S rRNA and Colonic Transcriptome Sequencin

In brief, the fecal samples were sent to Biomarker Technologies Co., Ltd. (Beijing, China) for 16S rRNA sequencing analysis. For colonic transcriptome sequencing, mice were anesthetized and executed by cervical dislocation, and colon tissues were dissected and collected from five mice in each group. Samples were sent to Biomarker Technologies Co., Ltd. (Beijing, China) for analysis. The detailed methods are shown in the Supplementary Materials and Methods.

### 2.7. Real-Time Quantitative PCR (qPCR)

Total RNA was extracted from colon tissue using TRIzol reagent (Life Technologies, Carlsbad, CA, USA). The extracted RNA was reverse transcribed into cDNA using the MonScriptTMRT III all-in-one Mix kit (Monad Biotech, Suzhou, China), and the MonAmpTM ChemoHS qPCR Mix (Monad Biotech, China) reagent was used for quantitative real-time fluorescent quantitative PCR amplification. The reaction conditions were 95 °C for 10 min, followed by 95 °C for 10 s, 60 °C for 20 s, and 72 °C for 30 s, for a total of 40 cycles. The results were evaluated according to the exponential growth of the fluorescence signal, the quantitative cycle (Cq) value, and the dissolution curve. β-actin was used as a control gene using the 2^−ΔΔCt^ method. The primer sequences are shown in Supplementary Table S2.

### 2.8. Protein Separation and Western Blot Analysis

Colon and ovarian tissues were milled by adding RIPA buffer (Beijing Solarbio Science & Technology Co., Ltd., Beijing, China) containing 1% PMSF (Solarbio, Beijing, China). Afterward, the samples were lysed on ice for 30 min. After lysis, the mixture was centrifuged at 16,000× *g* for 10 min to collect the protein-containing supernatant. Briefly, western blot experiments were performed as described previously [34]. The antibodies used in the study are shown in Supplementary Table S3. The detailed steps of western blot are shown in the Supplementary Materials and Methods.

### 2.9. Oocyte Collection and In Vitro Maturation

On the 8th day after DSS treatment, germinal vesicle (GV)-stage oocytes were collected from the bilateral ovaries of the mice under a stereomicroscope. The collected oocytes were placed in drops consisting of 30 μL of M16 medium covered with mineral oil and were incubated at 37 °C and 5% CO_2_ in an incubator for 12 h for maturation.

### 2.10. Reactive Oxygen Species (ROS) and Mitochondrial Membrane Potential (MMP, ΔΨm) Assays

To measure the level of ROS in oocytes, GV-stage oocytes were incubated in M16 containing 10 μM DCFHDA (Invitrogen, Rochester, NY, USA) for 30 min at 37 °C in an incubator with 5% CO_2_. Oocytes were then washed three times in PBS-PVA and photographed using a fluorescence microscope (Nikon, Tokyo, Japan). To detect the MMP level, MII-stage oocytes were placed into 2 μM 5,5′,6,6′-tetrachloro-1,1′,3,3′-tetraethylbenzimidazolyl carbocyanine iodide dye (Beyotime, Shanghai, China) containing PBS-PVA for 30 min at 37 °C in an incubator with 5% CO_2_. After washing with PBS-PVA three times, red/green fluorescence signals were captured using fluorescence microscopy. Images were analyzed for fluorescence intensity using ImageJ software.

### 2.11. Determination of ATP Levels

ATP levels were detected using an Enhanced ATP Assay Kit (Beyotime, China) according to the product instructions. Standard reaction solutions were prepared prior to measurement according to the manufacturer’s instructions. Briefly, 80 μL of lysate containing 50 oocytes was added to each well of a 96-well plate, followed by ultrasonic disruption, and the supernatant was taken as the sample to be tested. Then, the prepared ATP detection working solution was added. The optical detection value of the sample in the well was assayed using a microplate reader (Tecan, Mannedorf, Switzerland) with a standard curve for analysis.

### 2.12. In Vitro Fertilization (IVF)

The epididymal tail and vas deferens were removed from 10-week-old male mice, quickly placed in 200 μL of TYH medium (Nanjing Aibei Biotechnology Co., Ltd., Nanjing, China), and scratched to allow the sperm to flow out. The spermatozoa were incubated for 1 h at 37 °C in 5% CO_2_. The previously collected MII-stage oocytes were placed in human tubal fluid (Nanjing Aibei Biotechnology Co., Ltd., China), and 5 μL of sperm from the sperm capacitation fluid was added to the fertilization dish using a pipette. After 6 h of fertilization, zygotes were transferred from the fertilization droplet into the KSOM droplet. After 24 h of fertilization, the number of 2-cell-stage cells was observed and recorded; then, they were transferred into a new 50-μL KSOM culture medium droplet, and the medium was not changed for the next 48 h.

### 2.13. Follicle Count

The ovaries of the mice were sectioned and stained as described above. Follicle counts were performed on both ovaries according to a previous method [25]. All ovaries were fixed in 4% buffered paraformaldehyde, embedded in a paraffin block, and then cut into 4-µm sections serially. To avoid duplicate counting of follicles in the same field of view, a slice was selected every 25 µm for follicle counting. Each ovary required 10 sections to count the sum of follicles at all levels: (1) primordial follicle: surrounded by a single layer of flat granular cells; (2) primary follicle: surrounded by a single layer of cubic granular cells; (3) secondary follicle: more than two layers of cubic granular cells wrapped; (4) antral follicle: multilayer cubic granulosa cells are wrapped with an antrum; (5) atretic follicles: the nucleus of the oocytes shrinks, chromosomes and cytosols are dissolved, and the granule cell layer is reduced.

### 2.14. Statistical Analysis

All data are shown as the mean ± standard deviation (SD). A *t* test was used to compare the data from the two groups. One-way analysis of variance (ANOVA) was used to analyze differences between three or more groups. * *p* < 0.05 and ** *p* < 0.01 were considered statistically significant. All statistical analyses were performed using SPSS software (version 21.0, IBM, Chicago, IL, USA). The number of samples used (n) in different experiments are shown in the figure legends.

## 3. Results

### 3.1. DSS-Induced Colitis in Mice

**Figure 1:**
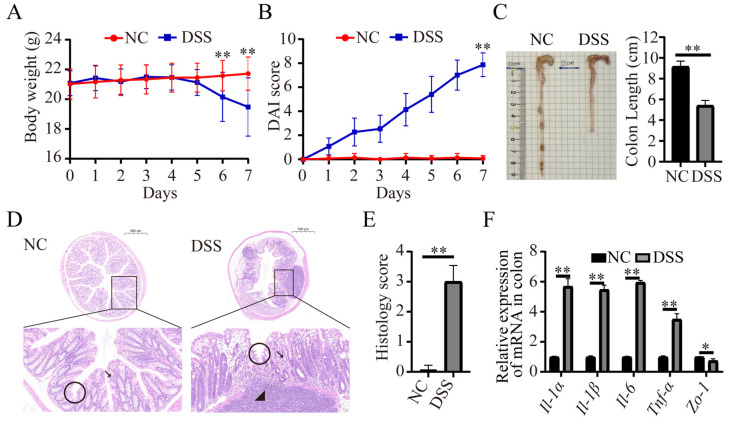
2.5% DSS induced acute colitis in mice. (**A**) DSS treatment reduced the body weight of mice (n = 15 for each group). (**B**) DAI score changes in mice between the NC and DSS groups (n = 15 for each group). (**C**) Representative image of the colon in mice with or without DSS treatment at day 7 (n = 15 for each group). DSS treatment significantly reduced the length of the colon (n = 15 for each group). (**D**) HE staining of colon tissue sections. The circles are located at crypts, arrows point to goblet cells, and triangles are located at inflammatory cells. Bar = 500 μm. (**E**) Histology score of mice in the NC and DSS groups. (**F**) The mRNA levels of inflammatory factors and tight junction proteins in colonic tissues. * *p* < 0.05; ** *p* < 0.01.

As shown in Figure 1A, the body weight of mice in the DSS group was significantly lower compared to that of the NC group on day 6 (NC group, 21.58 ± 1.01 g, n = 15; DSS group, 20.15 ± 1.66 g, n = 15, *p* < 0.01) and day 7 (NC group, 21.71 ± 1.12 g, n = 15; DSS group, 19.48 ± 1.66 g, n = 15, *p* < 0.01) (Figure 1A). On day 7, the DAI index of the DSS group was significantly higher than that of the NC group (NC group, 0.07 ± 0.26, n = 15; DSS group, 7.87 ± 0.99, n = 15, *p* < 0.01, Figure 1B). After the DSS treatment, colonic tissues were separated, and the length was measured. The colonic length of mice in the DSS group was significantly shorter (NC group, 9.18 ± 0.58 cm, n = 15; DSS group, 5.42 ± 0.49 cm, n = 15, *p* < 0.01, Figure 1C). HE staining of colon tissue showed that DSS group mice had increased inflammatory cell infiltration, severe crypt damage, and goblet cell loss (Figure 1D). The pathology score of the DSS group was significantly higher than that of the control group (NC group, 0.06 ± 0.13 cm, n = 5; DSS group, 2.74 ± 0.48 cm, n = 5, *p* < 0.01, Figure 1E). In addition, the mRNA levels of the inflammatory cytokines *Il-1α*, *Il-1β*, *Il-6*, and *Tnf-α* were significantly higher in the DSS group (*p* < 0.01, Figure 1F). The mRNA levels of *Zo-1* were found to be significantly lower in the DSS group by measuring the mRNA levels of Zo-1 in colonic tissues (*p* < 0.05, Figure 1F). The above results indicated that the mouse colitis model was successfully constructed after 7 days of consuming 2.5% DSS drinking water.

### 3.2. DSS Treatment Causes Dysbiosis of the Intestinal Flora

**Figure 2:**
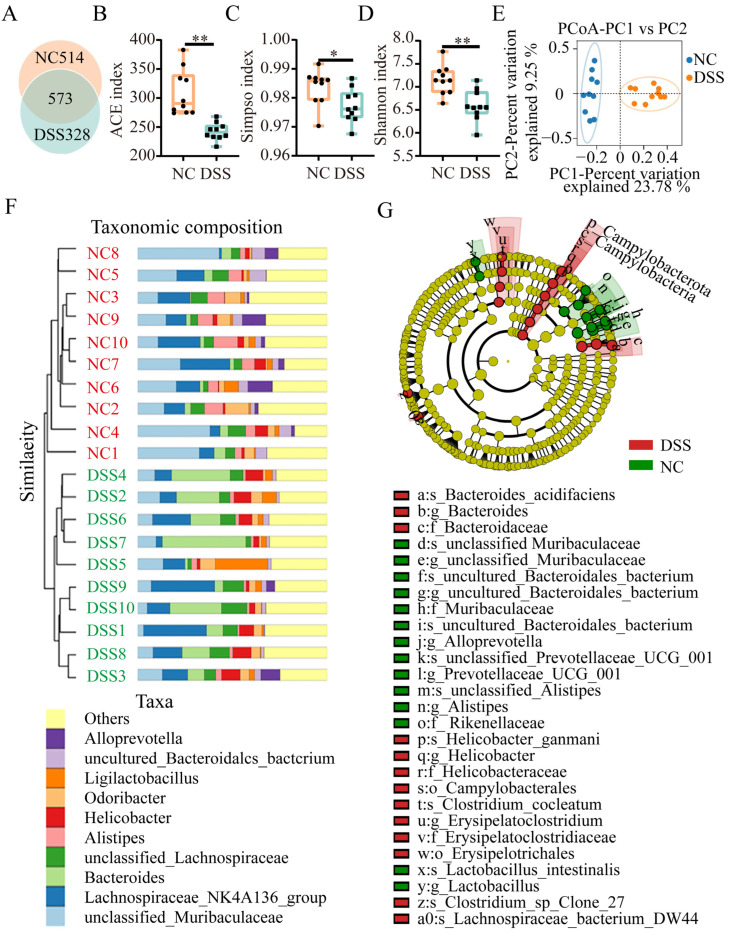
Effects of DSS treatment on the intestinal microbial diversity and composition in mice. (**A**) Venn diagram showing the OTU distribution between the groups. (**B**) ACE index changes in mice between the NC and DSS groups. (**C**) Simpson’s index changes in mice between the NC and DSS groups. (**D**) Shannon index changes in mice between the NC and DSS groups. (**E**) PCoA plot of the intestinal flora from mice in the NC and DSS group. (**F**) Relative abundances of the top 10 bacterial genera between the NC and DSS groups. Each color represents a genus, and the length of the patch represents the relative abundance ratio of all the bacterial communities in each sample. The other genera are merged into an “others” category. (**G**) Evolutionary branching plot of LEfSe analysis. Flora names are indicated with the order number letter (“a” to “a0”), abbreviation of taxonomy (p: Phylum; c: Class; o: Order; f: Family; g: Genus; and s: Species), and detailed name. * *p* < 0.05; ** *p* < 0.01.

A Venn diagram was used to identify common and characteristic taxa in different groups, and the results showed that the NC and DSS groups shared 573 OTUs; the number of OTUs specific to the NC and DSS groups was 514 and 328, respectively (Figure 2A). Compared with those of the NC group, the ACE (*p* < 0.01), Shannon (*p* < 0.05), and Simpson (*p* < 0.01) indices of the DSS group were significantly decreased (Figure 2B–D). PCoA showed that the flora composition of the NC group was different from that of the DSS group (Figure 2E). Hierarchical cluster analysis showed that the microbial community structure and composition in the NC group and the DSS group were significantly different at the genus level. *unclassified_Muribaculaceae*, *Lachnospiraceae_NK4A136_group*, Bacteroides, *unclassified_Lachnospiraceae*, *Alistipes*, *Helicobacter*, *Odoribacter*, *Ligilactobacillus*, *uncultured_Bacteroidalcs_bactcrium*, and *Alloprevotella* were the 10 most dominantly abundant bacteria in the NC and DSS groups (Figure 2F). DSS treatment altered the abundance of the intestinal flora at the phylum, family, genus, and species levels (Supplementary Figure S1). Moreover, the influence of the flora at different taxonomic levels with significant differences in abundance and composition was evaluated according to LEfSe analysis. At the genus level, *unclassified_Muribaculaceae*, *uncultured_Bacteroidales_bacterium*, *Alloprevotella*, *Prevotellaceae_UCG_001*, *Alistipes*, *Lactobacillus*, *Bacteroides*, *Helicobacter*, and *Erysipelatoclostridium* had the greatest changes in abundance and composition, which had great impacts on the entire colonic microbiota community. The influential flora with significant changes in abundance and composition at other taxonomic levels are shown in Figure 2G.

### 3.3. DSS Treatment Changes the Gene Expression in the Colon

**Figure 3:**
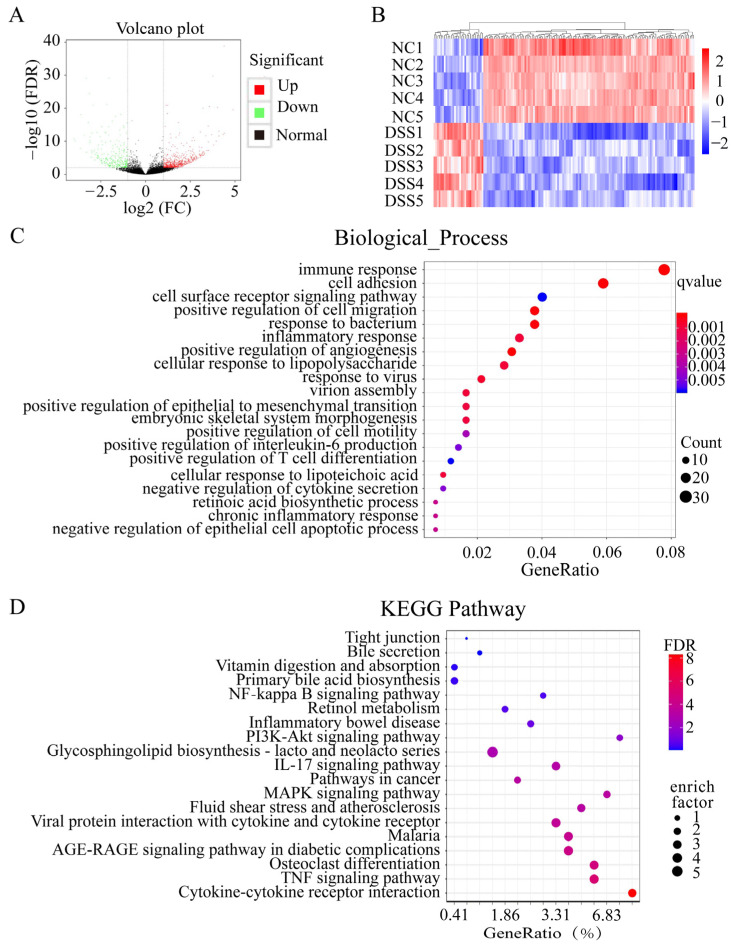
Differential gene GO and KEGG pathway analysis. (**A**) Distribution of all DEGs shown in a volcano map. (**B**) Heatmap showing the differentially expressed genes in common in these two comparisons. (**C**) GO enrichment analysis of DEGs. (**D**) KEGG enrichment analysis of DEGs.

There were 903 differentially expressed genes in the colon of DSS-induced colitis mice compared with normal mice. A total of 643 genes were significantly upregulated, while 260 genes were significantly downregulated (Figure 3A and Supplementary Table S4). There were differentially expressed gene hotspots in the NC group and the DSS group (Figure 3B). The differentially expressed genes were subjected to GO functional annotation analysis (Figure 3C). GO-enriched biological processes included immune response, inflammatory response, and cell surface receptor signaling pathway, in addition to retinoic acid biosynthesis. Next, KEGG pathway enrichment analysis showed that these DEGs were involved in 20 pathways, including the NF-kappa B signaling pathway, TNF signaling pathway, and inflammatory bowel disease. Specifically, bile secretion, primary bile acid biosynthesis, vitamin digestion and absorption, and retinol metabolism were also involved (Figure 3D).

### 3.4. Combined Analysis of the Gut Microbiota and Transcriptome

**Figure 4:**
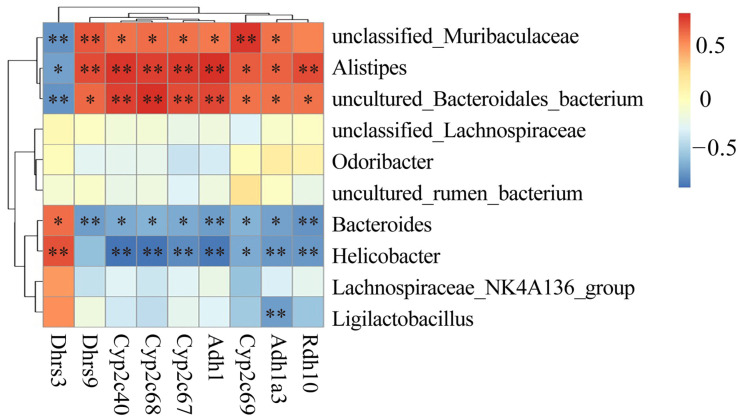
Correlation analysis between differentially expressed genes and flora for vitamin A metabolism. * *p* < 0.05; ** *p* < 0.01.

Based on the GO enrichment and KEGG enrichment results, we investigated whether the absorption and metabolism of vitamin A were related to the intestinal flora. Correlation analysis between differentially expressed genes and flora enriched for metabolism and the absorption of vitamin A was performed at the genus level using Spearman analysis (Figure 4). The results showed that genes related to the metabolic absorption of vitamin A (*Rdh10*, *Adh1a3*, *Adh1*, *Cyp26c67*, *Cyp26c68*, *Cyp26c40*, and *Dhrs9*) were positively correlated with *unclassified_Muribaculaceae*, *Alistipes*, and *uncultured_Bacteridales_bacterium*, while they were negatively correlated with *Helicobacter*, *Bacteroides*, and *Ligilactobacillus*. *Dhrs3* was positively correlated with *Helicobacter* and *Bacteroides*, while it was negatively correlated with *unclassified_Muribaculaceae*, *Alistipes*, and *uncultured_Bacteridales_bacterium*. These results suggest that ulcerative colitis-induced dysbiosis of the intestinal flora may trigger metabolism-related genetic alterations in vitamin A in the mouse colon.

### 3.5. DSS-Induced Colitis Impairs Ovarian Function in Mice

**Figure 5:**
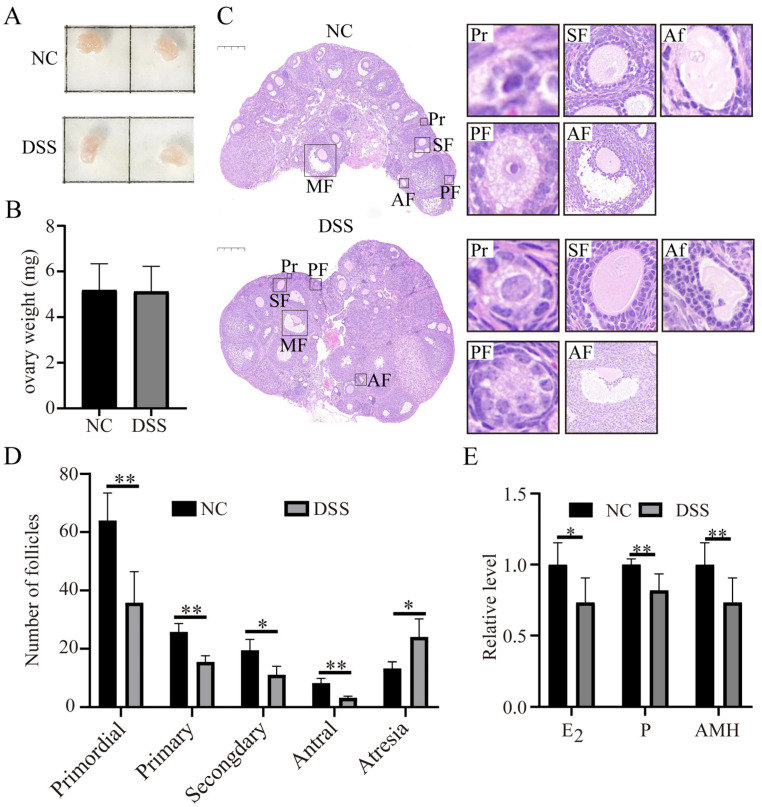
Effects of DSS-induced colitis on ovarian function in mice. (**A**) General ovary morphology with or without DSS treatment. (**B**) Ovary weight of mice in NC group and DSS group (n = 15 for each group). (**C**) Representative HE sections of ovaries in NC group and DSS group (n = 4 for each group). Bar = 200 μm. Primordial follicles (Pr), primary follicles (PF), secondary follicles (SF), antral follicles (AF), and atretic follicles (Af). (**D**) Follicle counts at all levels: primordial follicles, primary follicles, secondary follicles, antral follicles, and atretic follicles. (**E**) The relative levels of E_2_, P, and AMH in serum of mice in NC group and DSS group. * *p* < 0.05; ** *p* < 0.01.

To investigate the effect of colitis on ovarian function in mice, bilateral ovaries were collected (Figure 5A), and there were no significant differences in general observations or weight between normal and DSS-treated mice (NC group, 5.18 ± 1.16 mg, n = 15; DSS group, 5.12 ± 1.09 mg, n = 15, Figure 5B). The numbers of primordial follicles (NC group, 64.00 ± 9.42; DSS group, 35.75 ± 10.78, *p* < 0.01), primary follicles (NC group, 25.75 ± 2.87; DSS group, 15.5 ± 2.08, *p* < 0.05), secondary follicles (NC group, 19.5 ± 3.70; DSS group, 3.25 ± 0.50, *p* < 0.05), and antral follicles (NC group,13.25 ± 2.21; DSS group, 24 ± 6.21, *p* < 0.05) in the DSS group was significantly reduced compared with those in the NC group (Figure 5C,D). The number of atretic follicles was significantly increased in the DSS group (NC group, 13.25 ± 2.22; DSS group, 24 ± 6.21, *p* < 0.01). Furthermore, the levels of serum E_2_ (*p* < 0.05), P (*p* < 0.01) and AMH (*p* < 0.01) were significantly lower in the DSS group than in the NC group (Figure 5E). These findings suggest that DSS-induced ulcerative colitis decreases ovarian reserve and impairs ovarian endocrine function.

### 3.6. Colitis Reduced the Quality of Mouse Oocytes

**Figure 6:**
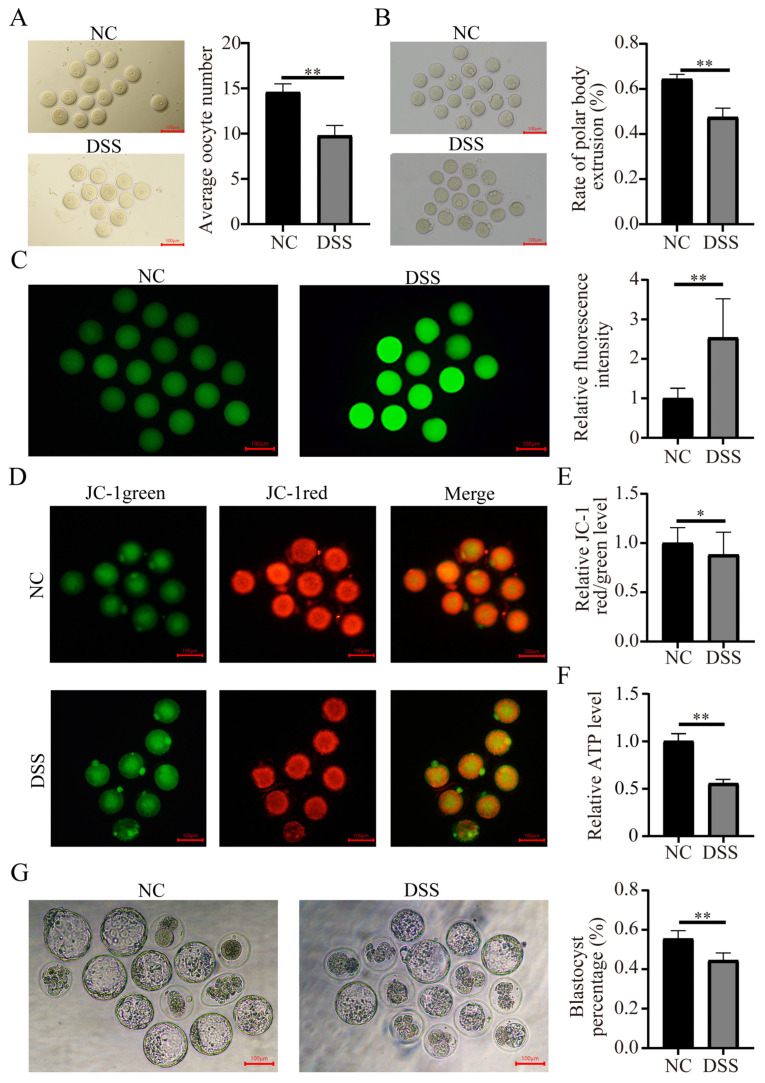
Effects of DSS-induced ulcerative colitis on oocyte quality. (**A**) Representative image of GV-stage oocytes collected from the ovaries of mice in the NC and DSS groups. Bar = 100 μm. (**B**) Representative picture of oocytes developing from the GV to MII stage and the first polar body extrusion rate of oocytes in the NC group and DSS group. Bar = 100 μm. (**C**) Representative images of ROS signals in GV-stage oocytes of the NC and DSS groups. Changes in the relative DCFHDA fluorescence intensity levels in GV-stage oocytes. (**D**) Representative JC-1 staining images of MII-stage oocytes in the NC and DSS groups. (**E**) Relative fluorescence levels of JC-1 in MII-stage oocytes. (**F**) Relative change of ATP levels in MII-stage oocytes. (**G**) Images of oocytes developing to blastocysts after IVF. Ratio of oocytes developing to blastocysts after IVF in the NC and DSS groups. * *p* < 0.05; ** *p* < 0.01.

Compared with the NC group, the number of oocytes in the DSS group was significantly reduced. (NC group, 14.00 ± 1.00; DSS group, 9.33 ± 1.53, *p* < 0.01, Figure 6A). After culture for 12 h in vitro, most oocytes in the DSS-treated mice did not extrude the first polar body (NC, 63.00 ± 1.91%; DSS, 47.02 ± 3.90%, *p* < 0.01, Figure 6B). The fluorescence intensity of DCFHDA in oocytes of the DSS group was significantly higher than that of NC group. (2.03 ± 0.30-fold, *p* < 0.01, Figure 6C). The MMP level of oocytes in DSS-treated mice was significantly lower than that of normal mice (0.77 ± 0.16-fold, *p* < 0.01, Figure 6D,E). The level of ATP was significantly lower in DSS-treated mice (0.42 ± 0.03-fold, *p* < 0.01, Figure 6F). In addition, the blastocyst rate of oocytes fertilized in vitro derived from DSS-treated mice was significantly lower (NC, 55.50 ± 4.05%; DSS, 44.49 ± 3.75%, *p* < 0.01, Figure 6G) than that of oocytes from normal mice. These results suggest that colitis impairs the quality of oocytes and reduces the embryo development capacity.

### 3.7. DSS-Induced Colitis Induces Abnormal Vitamin A Metabolism and Reduces Steroid Hormone Synthesis

**Figure 7:**
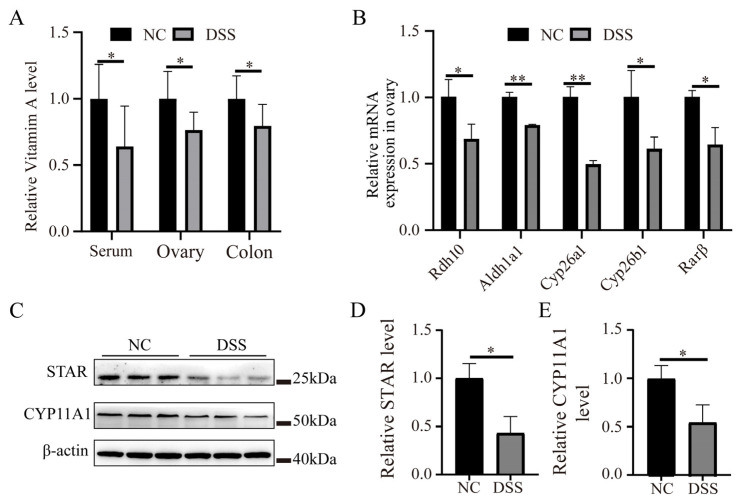
Effects of DSS-induced colitis on the expression of vitamin A metabolism- and steroid hormone synthesis-related proteins in the ovary. (**A**) Relative content of vitamin A in the serum, colon, and ovary. (**B**) The mRNA level of vitamin A metabolism-related genes in ovarian tissue. (**C**) Western blot detection of STAR and CYP11A1 proteins in the ovary of mice with or without DSS treatment. (**D**) Relative expression levels of STAR protein. (**E**) Relative expression levels of CYP11A1 protein. * *p* < 0.05; ** *p* < 0.01.

The results of the gut microbial and transcriptomic analyses suggested that DSS-induced ulcerative colitis triggered impaired absorption of vitamin A. Vitamin A plays an important role in the regulation of animal reproduction. Therefore, we examined the level of vitamin A in the serum, colon, and ovary. The results showed that the levels of vitamin A in the serum, ovary, and colon were significantly lower in the DSS-treated group than in the NC group (*p* < 0.05; Figure 7A). The mRNA expression levels of the vitamin A metabolism-related genes *Rdh10*, *Aldh1a1*, *Cyp26a1*, *Cyp26b1*, and *Rarβ* in the ovaries of DSS-treated mice were significantly decreased (Figure 7B), indicating that the vitamin A metabolism process in the ovary might be abnormal. In addition, the protein levels of the STAR and CYP11A1 in the ovary were significantly decreased to 0.43 ± 0.17-fold and 0.56 ± 0.18-fold compared with levels in the NC group, respectively (Figure 7D,E, *p* < 0.05).

## 4. Discussion

DSS-induced colitis in mice shows symptoms similar to mammalian enteritis and can be used as a model for animal enteritis studies [35]. In this study, DSS was used to construct an ulcerative colitis model to explore the effects of colitis on ovarian function in mice. After 7 days of DSS consumption in drinking water, mice in the DSS-treated group showed weight loss, bloody stools, shortened colon length, and an increased DAI score. HE staining of the colon showed that inflammatory cell infiltration and loss of goblet cells occurred in the DSS group. The mRNA levels of the proinflammatory factors *Il1α*, *Il-1β*, *Il-6*, and *Tnf-α* were significantly increased in the DSS group, while the intestinal barrier system-related gene *Zo-1* was significantly decreased. The destruction of the intestinal barrier causes the invasion of pathogens, which induce oxidative stress, thereby enhancing the intestinal permeability and causing intestinal barrier dysfunction [36]. These results indicate that the ulcerative colitis model was successfully constructed [20,37]. In addition, we found abnormalities in the intestinal barrier system and microbial community structure and composition after DSS treatment. From the current results, we cannot determine whether DSS can directly affect the related functions, including vitamin A absorption, without affecting the microbiota. However, compared with the normal intestinal physiological status, DSS treatment is an important trigger for the dysbiosis of intestinal microbiota composition, leading to inflammation and impaired vitamin A absorption.

Dysbiosis of the intestinal flora is an important feature in the development of inflammatory bowel disease [38]. DSS treatment significantly reduced the α and β diversity of intestinal microorganisms, indicating that DSS-induced colitis reduced the diversity and richness of the intestinal flora and changed the composition of the intestinal flora, which in turn caused dysbiosis of the intestinal flora. The intestinal flora characteristics of patients with inflammatory bowel disease are different from those of healthy individuals [39]. LEfSe analysis was used to compare NC and DSS-treated mice, and internal subgroup comparison analysis was conducted to identify species with significant differences in abundance between the two groups at different taxonomic levels. *Erysipelatoclostridium* was found to be positively correlated with the DAI scores, pathological score, TNF-α, and IL-1β [40]. *Bacteroides* have been shown to be associated with the deterioration of inflammatory bowel disease [41,42,43,44,45]. In contrast, *Muribaculaceae* and *Lactobacillus* can produce succinate, acetate, propionate [46], and butyrate [47], which alleviate inflammatory bowel disease by reducing the level of intestinal inflammatory factors and restoring the balance of the intestinal microbiota [48,49]. The changes in the abundances of *Rikenellaceae* and *Alistipes* in this study are also consistent with the results that the use of *Lactobacillus* strains and compounds can promote the recovery of intestinal tight junctions, mucus thickness, and intestinal flora stability in a DSS-induced colitis model [50,51,52]. In addition, the intestinal flora may be involved in the metabolic absorption of host vitamin A [53]. When animals are vitamin A deficient, the abundance of *Helicobacter increases* [54], while the abundances of *Muribaculaceae*, *Roseburia*, and *Lactobacillus* increase when animals are vitamin A sufficient [55,56,57]. Combined with the results related to this study, DSS-induced ulcerative colitis resulted in a decrease in SCFA-producing bacterial genera. This change disrupts the homeostasis of the colonic internal environment and may affect the absorption and metabolism of vitamin A in the host while driving the progression of colitis.

After treatment with DSS, a total of 903 DEGs were found in the colon of mice. KEGG pathway enrichment analysis showed that these DEGs were enriched in inflammation-related pathways, including cytokine—cytokine receptor interaction, the TNF signaling pathway, the PI3K-Akt signaling pathway, inflammatory bowel disease, the NF-kappaB signaling pathway, and tight junctions. This result suggests that DSS treatment may enhance the phosphorylation of PI3K/Akt and that phosphorylated Akt (p-Akt) activates NF-κB by enhancing the phosphorylation of IκB [58], which in turn promotes the synthesis of the inflammatory cytokines IL-6, IL-1β, and TNF-α [59]. DSS-induced colitis decreases the expression of tight junction proteins in mice, which leads to disruption of the intestinal barrier [60]. These results indicate that a damaged gut barrier might lead to increased oxidative stress due to toxic substances and proteins such as LPS from streptococcus spp. Interestingly, we also found that many DEGs were involved in bile secretion and primary bile acid biosynthesis. Related studies have found that the relative abundances of cholic acid (CA), lithocholic acid (LCA), and taurodeoxycholic acid (TUDCA) in DSS-induced colitis mice were significantly reduced, and the homeostasis of bile acids was destroyed [18,19]. The absorption of vitamin A depends on bile acids; dysregulation of bile acids can disrupt absorption [14,61]. In contrast, vitamin A supplementation can alleviate colitis [62,63]. These results suggest that DSS-induced colitis causes intestinal barrier dysfunction and mucosal inflammation by damaging the epithelial barrier, allowing dysbiosis and translocation of the flora, thereby potentially causing bile acid metabolism disorders and interfering with vitamin A absorption.

The ovary is an important reproductive organ for ovulation and the secretion of sex hormones in female animals. To investigate the effects of colitis on ovarian function, the morphology and weight of the bilateral ovaries of mice were first assessed. There was no difference in ovarian morphology or weight. However, the number of primordial follicles and antral follicles in the ovaries of colitis mice decreased, while the number of atresia follicles increased. This finding indicates that colitis leads to a significant decrease in ovarian reserve. At the same time, the level of serum AMH in the DSS group was significantly reduced, which was consistent with the decrease in follicle count in the DSS group. This result may be related to the fact that AMH maintains the number of primordial follicles in the follicular pool by inhibiting the excessive activation of primordial follicles [64,65,66]. This function supports the conclusion that DSS-induced inflammation led to a significant reduction in ovarian reserve in this study because AMH is mainly secreted by granulosa cells of the antral follicles [67,68].

In addition to oocyte generation, the ovary is also responsible for producing steroid hormones. Proper levels of steroid hormones play an important role in the reproductive health of female animals. Estrogen and progesterone are the most important steroid hormones in the ovary and are mainly produced by granulosa cells prior to ovulation [69]. In this study, a significant decrease in both estrogen and progesterone in the serum levels was found in DSS-treated mice. This finding suggests that DSS-induced ulcerative colitis leads to estrogen deficiency, which in turn potentially affects the number of primordial and primary follicles in the ovary. This response may be due to the estrogen reduction prematurely activating primordial follicles in the ovary [70]. Reduced estrogen also leads to impaired follicular development and premature atresia [71]. This implies that acute colitis may reduce estrogen by inducing the apoptosis of granulosa cells in a short period of time and may lead to follicular atresia [72]. In addition, in vitro studies have shown that inhibition of progesterone production during oocyte maturation greatly reduces the percentage of MII-stage oocytes [73]. Progesterone can influence oocyte quality through its effect on the development of the dominant follicle, and it plays an important role in maintaining pregnancy and increasing the embryo implantation rate during ovulatory follicle development [74,75]. This information suggests that DSS-induced ulcerative colitis leads to a decrease in progesterone levels and damages the development of antral and mature follicles.

In this study, DSS-induced colitis triggered ovarian dysfunction, resulting in reduced ovarian reserve and impaired endocrine function, while the quality of oocytes was greatly reduced. After DSS treatment, the in vitro maturation rate of oocytes decreased significantly. The levels of ROS and ATP in oocytes of the DSS group were significantly increased. This result implies that oocytes from individuals with DSS-induced colitis may undergo mitochondrial dysfunction, which ultimately reduces oocyte quality and hinders oocyte maturation [76,77]. Both MMP and ATP levels were significantly decreased in oocytes from the DSS-treated group, which supports our hypothesis that DSS-induced ulcerative colitis may cause oxidative stress in oocytes and reduce their fertilization potential [78,79,80]. IVF-related results also suggested that the oocyte-derived embryonic development ability of the DSS group was significantly lower than that of the NC group, implying that the potential adverse effects of DSS-induced ulcerative enteritis could persist at least until the blastocyst stage.

According to previous studies [16,17] and the combined transcriptome and intestinal flora analysis in this study, we hypothesized that impaired ovarian function is associated with the impaired absorption and utilization of vitamin A induced by ulcerative colitis. Adequate vitamin A is essential to maintain normal reproductive function. When vitamin A deficiency occurs in mammals, it affects the implantation of embryos [81]. Therefore, the levels of vitamin A in serum, colon, and ovary were determined; we found that vitamin A levels were significantly decreased in the DSS group. *Rdh10* encodes retinol dehydrogenase, which converts retinol to retinaldehyde [82]. *Adh1a3* encodes aldehyde dehydrogenase, which catalyzes the formation of retinoic acid [83]. *Dhrs3* and *Dhrs9* are responsible for catalyzing the biosynthesis of retinoic acid from retinal [84,85], while *Cyp2c68* and *Cyp2c40* are genes related to retinol metabolism [86]. Combined with the results that *Rdh10*, *Adh1a3*, *Adh1*, *Dhrs9*, *Cyp2c68*, and *Cyp2c40* were positively correlated with *Muribaculaceae*, *Alistipes*, and *uncultured_Bacteridales_bacterium*, while they were negatively correlated with *Helicobacter* and *Bacteroides*, it is suggested that the imbalance in the flora may cause abnormal transformation of vitamin A in the host. The mRNA levels of *Rdh10*, *Aldh1a1*, *Cyp26a1*, *Cyp26b1*, and *Rarβ* in the ovaries of the DSS group were significantly decreased, suggesting that the production and oxidation of retinoic acid were reduced. This finding also means that colitis can cause abnormal use and metabolism of vitamin A in the ovaries, which is related to the significant prolongation of the estrous cycle of mice and the reduction in oocyte maturation, fertilization, and blastocyst formation [87].

The result regarding the reduction in ovarian reserve in the DSS-treated mice is also similar to other studies in which vitamin A deficiency led to a decrease in the number of total follicles and the corpus luteum, an increase in atretic follicles, and a decrease in the number and quality of ovulated oocytes [88]. This finding may also be related to the fact that vitamin A is an antioxidant that prevents oxidative damage and improves oocyte maturation and quality by maintaining adequate levels of antioxidant compounds and endogenous enzymes [89]. This is also supported by the results showing elevated ROS in oocytes of the DSS-treated group of mice. Vitamin A is involved in the production of ovarian steroid hormones. Vitamin A deficiency decreases the steroidogenic activity of the gonads [90]. Retinoic acid, the active form of vitamin A, promotes STAR and p-STAR protein levels; increases *Cyp17*, *Cyp11A1* and *Star* mRNA expression; and increases the levels of pregnenolone and progesterone [24]. Progesterone and estradiol are steroid hormones, both of which are formed from cholesterol. First, cholesterol is used to synthesize pregnenolone. Then, pregnenolone is converted to progesterone. Pregnenolone can also be converted into androgens and then into estrogens [91]. During this process, STAR is thought to mediate the rapid increase in steroid hormone biosynthesis by facilitating the entry of cholesterol into the inner mitochondrial membrane and is the rate-limiting step in steroidogenesis [92]. CYP11A1 converts cholesterol to the steroid hormone precursor pregnenolone [93]. This study found that the levels of STAR and CYP11A1 in the ovaries of mice in the DSS group were significantly decreased, which was consistent with the results of decreased E_2_ and P levels. These results suggest that the DSS-induced disruption of the flora composition and colitis can affect ovarian function through abnormal vitamin A utilization and metabolism.

## 5. Conclusions

In summary, DSS-induced ulcerative colitis caused an imbalance in the intestinal flora. The altered intestinal flora was correlated with gene expression in the colon, which in turn caused impaired vitamin A absorption and metabolism. The abnormal metabolism of vitamin A in the ovaries impaired follicular development, decreased steroid hormone secretion, and caused a decrease in mouse oocyte quality. All related results supported our hypothesis that DSS-induced colitis and impaired vitamin A absorption reduced ovarian function. Our work expanded the female animal gonad–gut axis effects by coupling the intestinal flora and ovarian function, revealing that differences in the microbial community composition and abundance are important for ovarian physiology and function.

## Data Availability

The data presented in this study are available on request from the corresponding author.

## Supplementary Materials

The following supporting information can be downloaded at: https://www.mdpi.com/article/10.3390/nu15112425/s1, Supplementary Materials and Methods; Table S1: Sequencing quality of all samples; Evaluation criteria of Disease activity index (DAI); Table S2: Primer sequences; Table S3: Antibody information; Table S4: Information of all DEGs; Figure S1: Abundance changes of the intestinal flora at the phylum, family, genus, and species levels with or without DSS treatment.
