# Supplementary material for: Colitis-Mediated Dysbiosis of the Intestinal Flora and Impaired Vitamin A Absorption Reduce Ovarian Function in Mice
Source: Nutrients. 2023 May 23;15(11):2425. doi: 10.3390/nu15112425 (PMC10254821; doi:10.3390/nu15112425)
Supplement: Supplementary file 1 [file nutrients-15-02425-s001.zip › nutrients-2393417-supplementary/Supplementary Materials/Supplementary Figure S1.pdf]

A

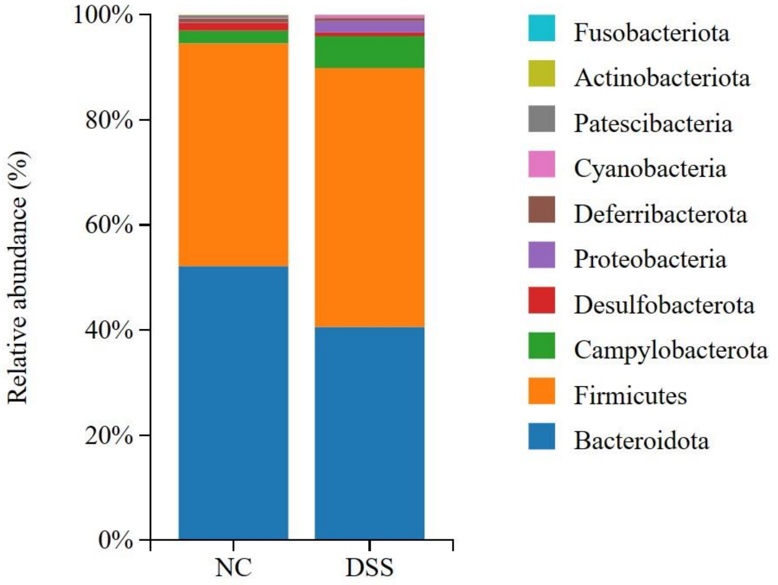

100%
80%
60%
40%
20%
0%
Relative abundance (%)
NC
DSS
Fusobacteriota
Actinobacteriota
Patescibacteria
Cyanobacteria
Deferribacterota
Proteobacteria
Desulfobacterota
Campylobacterota
Firmicutes
Bacteroidota

B

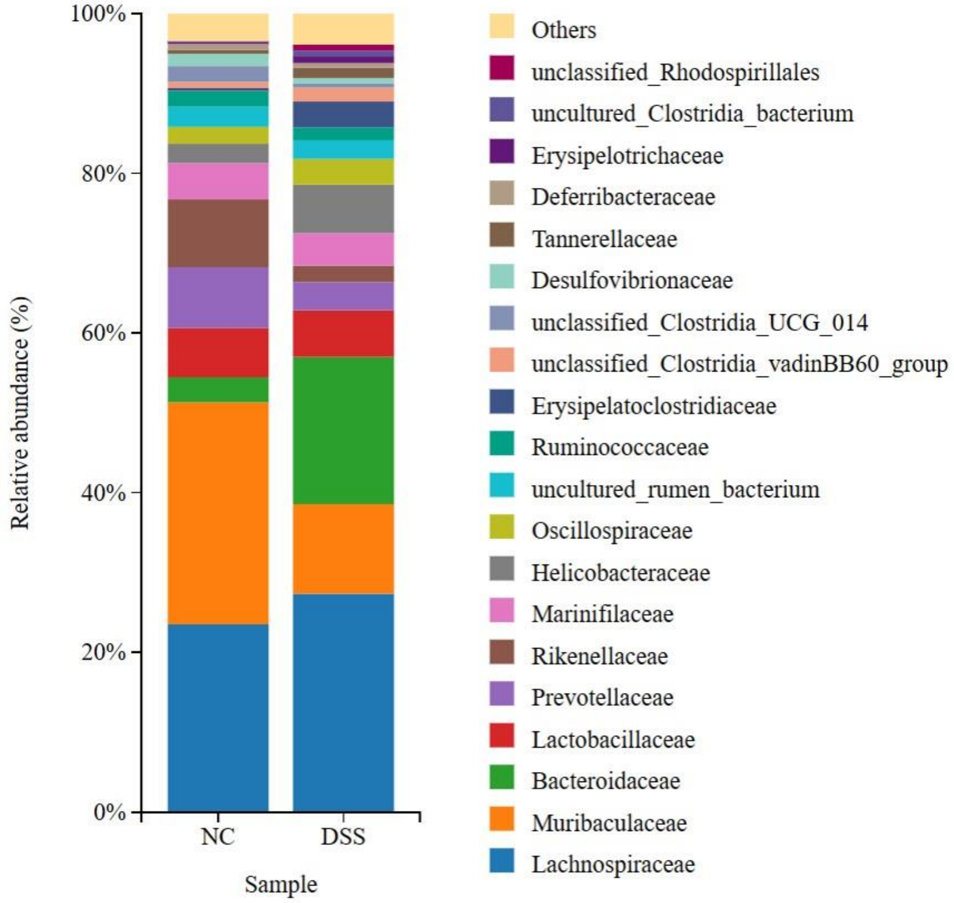

100%
80%
60%
40%
20%
0%
Relative abundance (%)
NC
DSS
Sample
Others
unclassified_Rhodospirillales
uncultured_Clostridia_bacterium
Erysipelotrichaceae
Deferribacteraceae
Tannerellaceae
Desulfovibrionaceae
unclassified_Clostridia_UCG_014
unclassified_Clostridia_vadinBB60_group
Erysipelatoclostridiaceae
Ruminococcaceae
uncultured_rumen_bacterium
Oscillospiraceae
Helicobacteraceae
Marinifilaceae
Rikenellaceae
Prevotellaceae
Lactobacillaceae
Bacteroidaceae
Muribaculaceae
Lachnospiraceae

C

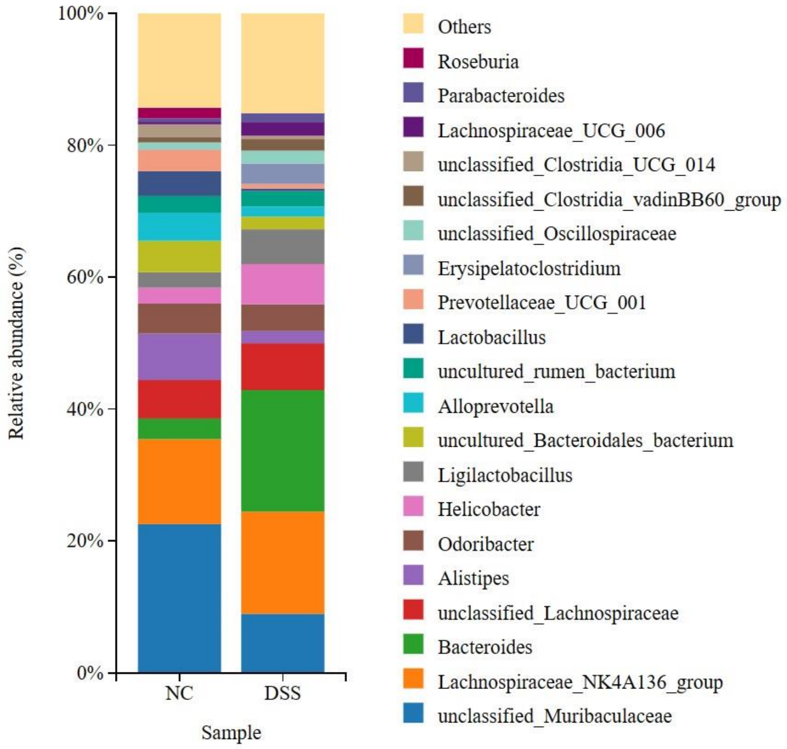

D

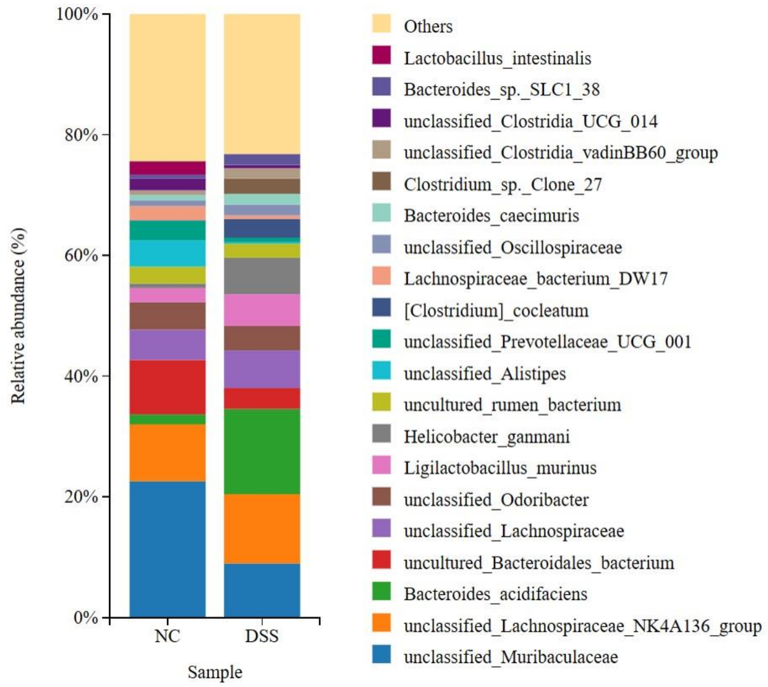

Supplementary Figure S1. Abundance changes of the intestinal flora at the phylum, family, genus, and species levels with or without DSS treatment. DSS treatment altered the abundance of the intestinal flora at the phylum (A), family (B), genus (C), and species (D) levels.
