# Supplementary material for: Colitis-Mediated Dysbiosis of the Intestinal Flora and Impaired Vitamin A Absorption Reduce Ovarian Function in Mice
Source: Nutrients. 2023 May 23;15(11):2425. doi: 10.3390/nu15112425 (PMC10254821; doi:10.3390/nu15112425)
Supplement: Supplementary file 1 [file nutrients-15-02425-s001.zip › nutrients-2393417-supplementary/Supplementary Materials/Supplementary Materials and Methods.pdf]

Supplementary Materials and Methods

1. 16S rRNA gene sequencing

First, total DNA was extracted from the samples using the PowerSoil® DNA Isolation kit according to the manufacturer's instructions. After extraction, the 16S full-length primer was designed according to the conserved region 27F-1492R for polymerase chain reaction (PCR) amplification of the target area (10 μL system, Solexa PCR). The synthesized specific primers (27F-1492R) were designed as follows: forward primer 27F: AGRGTTTGATYNTGGCTCAG; reverse primer 1492R: TASGGHTAC-CTTGTTASGACTT.

Subsequently, quality inspection was performed on the formed sequencing library, and processing, including barcode recognition, was performed on the high-quality circular consensus sequencing (CCS) sequence obtained. The generated optimization CCS was clustered at the level of 97% similarity (USEARCH, version 10.0), and its species classification was obtained based on the sequence composition of the operational taxonomic unit (OTU). The 16S Silva database and RDP Classifier platforms were used to analyze species annotation and taxonomy as well as the diversity of the gut microbiota. Alpha diversity analysis was performed to examine the species richness and diversity within a single sample, and the ACE, Chao, Shannon, and Simpson indices of each sample were calculated. The differences in the community composition and structure of different samples were compared through beta diversity analysis. Finally, linear discriminant analysis effect size (LEfSe) analysis was used to screen biomarkers that were significantly different between the groups (biomarker screening criteria: LDA score >4).

2. Colonic transcriptome sequencing

After the experiment, mice were anesthetized and executed by cervical disl ocation, and colon tissues were dissected and collected from five mice in each group. Total RNA was extracted using TRIzol reagent. The libraries were sequ enced on an Illumina NovaSeq platform. Transcriptome library preparation and sequencing services were provided by Biomarker Technologies Co., Ltd. (Beijin g, China). To ensure the accuracy of the subsequent analysis, reads containing connectors and low quality (including reads with >10% of N removed; and rea ds with more than 50% of the whole reads with the number of bases with a quality value Q ≤ 10 were removed) were removed, and the resulting highqual ity clean data were provided in FASTQ format. The information for the versio n of the reference genome is as follows: Mus_musculus.GRCm38_release95.gen ome.fa (https://jan2019.archive.ensembl.org/Mus_musculus/Info/Index). The raw r eads were further processed with a bioinformatics pipeline, the BMKCloud (htt ps://www.biocloud.net) online platform. Differential expression analysis of two c onditions/groups was performed using DESeq2. DESeq2 provides statistical rout ines for determining differential expression in digital gene expression data usin g a model based on the negative binomial distribution. The resulting P values

were adjusted using Benjamini and Hochberg's approach for controlling the fals e discovery rate. Genes with an adjusted p value < 0.01 and a fold change ≥ 2 found by DESeq2 were assigned as differentially expressed. The functions of differentially expressed genes, based on gene ontology (GO; http://geneontolog y.org) and Kyoto Encyclopedia of Genes and Genomes (KEGG; https://www.ke gg.jp), were enriched using the GOseq R package and KOBAS, and the correc ted $p < 0.05$.

3. Protein separation and western blot analysis

The samples were mixed with SDS–PAGE loading buffer (Solarbio, Beijing, China) containing 1% PMSF (Solarbio, Beijing, China) and incubated at 95°C for 10 min. Equal amounts of protein samples (20 μg) were separated by 10% SDS–PAGE (Epizyme Biomedical Technology Co., Ltd., Shanghai, China) and then transferred to polyvinylidene difluoride membranes (0.45 μm, Millipore, Bedford, MA, USA). Next, the membranes were transferred to a 5% BSA blocking solution at room temperature and blocked for 2 h. Next, the membranes were incubated with the corresponding primary antibody (dilution, 1:1000) at 4°C overnight. The membranes were rinsed 3 times with TBS-T buffer. The cells were incubated with horseradish peroxidase (HRP)-coupled anti-rabbit secondary antibody (dilution, 1:10,000) for 1 h at room temperature and then were washed three times with TBS-T buffer. Finally, a chemiluminescence imaging system (Monad Biotech, China) was used for image acquisition. The antibodies used in the study are shown in Supplementary Table S3.
