# Supplementary material for: Colitis-Mediated Dysbiosis of the Intestinal Flora and Impaired Vitamin A Absorption Reduce Ovarian Function in Mice
Source: Nutrients. 2023 May 23;15(11):2425. doi: 10.3390/nu15112425 (PMC10254821; doi:10.3390/nu15112425)
Supplement: Supplementary file 1 [file nutrients-15-02425-s001.zip › nutrients-2393417-supplementary/Supplementary Materials/Supplementary Table S1.pdf]

Supplementary Table S1. Evaluation criteria of Disease activity index (DAI) scores

DAI score	Weight loss (%)	Stool condition	Gross bleeding
0	None	Normal	None
1	< 5	Normal	None
2	5-10	Loose stools	Hemoccult positive
3	>10 and ≤ 20	Loose stools	Hemoccult positive
4	> 20	Diarrhea	Severe bleeding
