# Supplementary material for: Colitis-Mediated Dysbiosis of the Intestinal Flora and Impaired Vitamin A Absorption Reduce Ovarian Function in Mice
Source: Nutrients. 2023 May 23;15(11):2425. doi: 10.3390/nu15112425 (PMC10254821; doi:10.3390/nu15112425)
Supplement: Supplementary file 1 [file nutrients-15-02425-s001.zip › nutrients-2393417-supplementary/Supplementary Materials/Supplementary Table S2.pdf]

Supplementary Table S2. Primer sequences used in this study.

Genes	Accession Number	Primer Sequence (5‘to3’) [a]	Product length（bp）
β-actin	NM_007393.5	F: GGCTGTATTCCCCTCCATCG	154
		R: CCAGTTGGTAACAATGCCATGT	
Tnf-α	NM_001278601.1	F: CCTGGCCTCTCTACCTTGTTG	100
		R: AGCCTGGTCACCAAATCAGC	
Il-1α	NM_010554.4	F: GTATGCCTACTCGTCGGGAG	120
		R: GGCAACTCCTTCAGCAACAC	
Il-1β	NM_008361.4	F: GTCGCTCAGGGTCACAAGAA	155
		R: GTGCTGCCTAATGTCCCCTT	
Il-6	NM_031168.2	F: CTGCAAGAGACTTCCATCCAG	131
		R: AGTGGTATAGACAGGTCTGTTGG	
Zo-1	NM_001163574.1	F: GCTTTAGCGAACAGAAGGAGC	156
		R: TTCATTTTTCCGAGACTTCACCA	
Aldh1a1	NM_013467.4	F: AGGCCCTCAGATTGACAAGGAACA	135
		R: AACACTGTGGGCTGCACAAAGAAG	
Rdh10	NM_133832.3	F: ATGGTTCGCCACATCTACCG	72
		R: CTCCTCACCTTTTCCAGCTTGC	
Cyp26a1	NM_007811	F: GCACAAGCAGCGAAGAAGGTGAT	103
		R: ACTGCTCCAGACAACTGCTGACTT	
Cyp26b1	NM_175475	F: GGCGGCTACCGCACTGT	87
		R: TGTCTCGGATGCTATCATGACACT	
Rarβ	NM_001289762	F: GCACTGACGCCATAGTGGTA	88
		R: CACCATCTCCACTTCCTCCT	

[a] F: Forward primer; R: Reverse primer.
