# Supplementary material for: Colitis-Mediated Dysbiosis of the Intestinal Flora and Impaired Vitamin A Absorption Reduce Ovarian Function in Mice
Source: Nutrients. 2023 May 23;15(11):2425. doi: 10.3390/nu15112425 (PMC10254821; doi:10.3390/nu15112425)
Supplement: Supplementary file 1 [file nutrients-15-02425-s001.zip › nutrients-2393417-supplementary/Supplementary Materials/Supplementary Table S3.pdf]

Supplementary Table S3. Antibody information.

Primary antibodies

Antibody	Company [a]	Cat. Number
β-actin	Cell Signaling Technology	4970S
StAR	Abcam	ab133657
CYP11A1	Abcam	ab175408

Secondary antibodies

Antibody	Company [a]	Cat. Number
Anti-mouse IgG	Cell Signaling Technology	7076S
Anti-rabbit IgG	Cell Signaling Technology	7074S

[a] Cell Signaling Technology, Beverly, MA, USA.

Abcam, Cambridge, MA, USA
